# Identification of high-risk factors associated with mortality at 1-, 3-, and 5-year intervals in gastric cancer patients undergoing radical surgery and immunotherapy: an 8-year multicenter retrospective analysis

**DOI:** 10.3389/fcimb.2023.1207235

**Published:** 2023-05-31

**Authors:** Yuan Liu, Lanyu Wang, Wenyi Du, Yukang Huang, Yi Guo, Chen Song, Zhiqiang Tian, Sen Niu, Jiaheng Xie, Jinhui Liu, Chao Cheng, Wei Shen

**Affiliations:** ^1^ Department of General Surgery, Wuxi People’s Hospital Affiliated to Nanjing Medical University, Wuxi, China; ^2^ Department of Urology, Wuxi People’s Hospital Affiliated to Nanjing Medical University, Wuxi, China; ^3^ Department of General Practice, Shandong Provincial Hospital Affiliated to Shandong First Medical University, Jinan, Shandong, China; ^4^ The First Affiliated Hospital of Nanjing Medical University, Nanjing, China; ^5^ Department of Neurosurgery, Wuxi People’s Hospital Affiliated to Nanjing Medical University, Wuxi, China

**Keywords:** gastric tumor, gastrectomy, immunotherapy, *Helicobacter pylori*, risk factor, machine learning

## Abstract

**Background:**

Combining immunotherapy with surgical intervention is a prevailing and radical therapeutic strategy for individuals afflicted with gastric carcinoma; nonetheless, certain patients exhibit unfavorable prognoses even subsequent to this treatment regimen. This research endeavors to devise a machine learning algorithm to recognize risk factors with a high probability of inducing mortality among patients diagnosed with gastric cancer, both prior to and during their course of treatment.

**Methods:**

Within the purview of this investigation, a cohort of 1015 individuals with gastric cancer were incorporated, and 39 variables encompassing diverse features were recorded. To construct the models, we employed three distinct machine learning algorithms, specifically extreme gradient boosting (XGBoost), random forest (RF), and k-nearest neighbor algorithm (KNN). The models were subjected to internal validation through employment of the k-fold cross-validation technique, and subsequently, an external dataset was utilized to externally validate the models.

**Results:**

In comparison to other machine learning algorithms employed, the XGBoost algorithm demonstrated superior predictive capacity regarding the risk factors that affect mortality after combination therapy in gastric cancer patients for a duration of one year, three years, and five years posttreatment. The common risk factors that significantly impacted patient survival during the aforementioned time intervals were identified as advanced age, tumor invasion, tumor lymph node metastasis, tumor peripheral nerve invasion (PNI), multiple tumors, tumor size, carcinoembryonic antigen (CEA) level, carbohydrate antigen 125 (CA125) level, carbohydrate antigen 72-4 (CA72-4) level, and *H. pylori* infection.

**Conclusion:**

The XGBoost algorithm can assist clinicians in identifying pivotal prognostic factors that are of clinical significance and can contribute toward individualized patient monitoring and management.

## Introduction

Gastric cancer is among the most prevalent malignancies and serves as the primary cause of cancer-related deaths, with a particularly high incidence observed in developing countries (Maomao et al., 2022; Sekiguchi et al., 2022). Global epidemiological surveys on tumors have indicated that the incidence of gastric cancer is on the rise, concurrent with a shift in dietary habits (Morgan et al., 2022). Early diagnosis and prompt treatment hold critical importance in tumor management. Over the years, the advent of comprehensive therapeutic modalities such as immunotherapy and molecular targeted drugs has significantly improved the survival rates of patients with advanced gastric cancer (Li et al., 2021). However, a considerable fraction of tumor cells lack the requisite molecules that can interact with the immune system or exhibit immune evasion mechanisms, consequently resulting in immunotherapy failure. Furthermore, immunotherapy necessitates specific biomarkers or gene expression in patients, thus limiting its applicability to certain patient populations. The presence of side effects such as immune suppression or immune hyperactivation post immunotherapy further impedes its development. In contemporary clinical practice, clinicians frequently resort to combining advanced surgical techniques with immunotherapy to combat gastric cancer; notwithstanding, treatment failure may still occur, ultimately leading to patient mortality. This can be attributed to diverse factors, such as tumor type, stage, location and size, patient age, physical condition, and immune system status. Tumor cells may undergo mutation and evolve, resulting in increased tumor resistance to treatment (Kakinuma et al., 2021; Xiang et al., 2021; Shibata et al., 2022).

Clinicians commonly use their clinical experience and factors such as the patient’s medical history and presentation to assess the risk of death after combination therapy for gastric cancer. However, this method has limitations in terms of accuracy and subjectivity. Imaging tests such as CT and MRI are also used in diagnosis, but they increase the workload of medical staff and are financially burdensome for patients’ families. Additionally, some examination protocols are invasive and radioactive, which can cause harm to patients. Traditional regression models have been used, but they have poor discrimination and calibration ability (Niu et al., 2020). Artificial intelligence, particularly machine learning algorithms, can analyze and learn from large amounts of data to discover complex relationships and patterns between variables, enabling prediction of future disease occurrence (Wang et al., 2015). Compared to traditional prediction methods based on statistical methods and empirical rules, machine learning algorithms have stronger adaptive and generalization capabilities and can adapt to a wider and more complex data situation while avoiding errors introduced by researchers’ subjective factors and limitations of research methods. Liu et al. employed sophisticated data mining techniques to enhance the identification of prognostic risk factors in individuals diagnosed with early-stage gastric cancer, focusing specifically on non-invasive variables (Liu et al., 2018; Afrash et al., 2023). In this study, we analyzed clinical data from patients with gastric cancer and utilized machine learning algorithms to develop a prediction model for patient death after radical gastric cancer surgery combined with immunotherapy to improve the quality of postoperative survival.

## Materials and methods

### Study subjects

In this study, we used data from the clinical databases of the Affiliated Wuxi People’s Hospital of Nanjing Medical University, Wuxi Second People’s Hospital, and Shandong Provincial Hospital affiliated with Shandong First Medical University. The criteria for patient inclusion in this study were as follows: (1) adult patients aged 18 years and above but below 80 years of age; (2) patients who underwent a combination of radical gastric cancer surgery and immunotherapy; (3) the surgical team involved senior surgeons with the expertise to independently perform radical gastric cancer surgery; and (4) patients were diagnosed with gastric adenocarcinoma through postoperative pathology. Exclusion criteria for the case included the following: (1) patients presenting with coexisting malignancies; (2) patients diagnosed with gastric cancer with distant metastasis based on pathological examination or imaging studies; (3) patients diagnosed with severe cardiovascular or respiratory diseases; (4) patients with significant liver or kidney pathology; and (5) patients with incomplete case data, missing clinical information, or absent visits. All patients in the study were followed up for at least 5 years after surgery. This study was conducted in accordance with the Declaration of Helsinki and was approved by the Ethics Committee of the Affiliated Wuxi People’s Hospital of Nanjing Medical University, Wuxi Second People’s Hospital, and Shandong Provincial Hospital affiliated with Shandong First Medical University, with approval number KY22085.

### Diagnosis of *Helicobacter pylori* infection and determination of associated factors

The diagnosis of *H. pylori* infection was established using three criteria: first, through postoperative bacterial culture of gastric mucosa, duodenal mucosa, gastric fluid, and expiratory samples to confirm the presence of positive *H. pylori*; second, through postoperative HE staining of gastric mucosal tissue sections to determine the presence of positive *H. pylori*; and third, through postoperative confirmation of *H. pylori* infection by means of urea breath test (UBT), fecal antigen test, and endoscopy active infection. The patient fulfilled all three criteria and was ultimately diagnosed with *H. pylori* infection (Hou et al., 2020). In this study, clinicians employed PD-1/PD-L1 checkpoint inhibitors for the immunotherapeutic treatment of patients.

### Study design and data collection

Clinical information of the patients was evaluated, including demographic characteristics, basic clinical features, basic medical history, laboratory test indices before and during combination therapy, tumor characteristics, and intraoperative information of the patients. All laboratory tests conducted prior to the combination therapy were collected within 24 hours of the day, which included the measurement of albumin (ALB) levels. All laboratory tests conducted after the combination therapy were collected within 48 hours and included the patient’s *H. pylori* infection status, as well as the levels of carcinoembryonic antigen (CEA), carbohydrate antigen 19-9 (CA19-9), carbohydrate antigen 72-4 (CA72-4), carbohydrate antigen 125 (CA125), neutrophil-to-lymphocyte ratio (NLR), procalcitonin (PCT), C-reactive protein (CRP), and serum amyloid A (SAA). Demographic information included sex, age, body mass index (BMI), and history of smoking and alcohol abuse. Basic clinical features comprised the American Society of Anesthesiologists physical status classification (ASA score), Nutrition Risk Screening 2002 (NRS2002) score, history of surgery, family history, history of adjuvant chemotherapy, and history of adjuvant radiotherapy. Medical history included anemia, diabetes mellitus, hypertension, hyperlipidemia, and coronary heart disease (CHD). The study included tumor characteristics such as tumor T-stage, N-stage, peripheral nerve invasion (PNI), tumor size, and tumor number, as well as intraoperative variables such as the surgical approach, type of surgery, number of intraoperative lymph node dissections, anastomosis, type of anastomosis, and whether the surgery was performed as an emergency. The outcome indicators for this study were patient mortality rates at one, three, and five years.

### Statistical analysis

Continuous variables were presented using medians and interquartile ranges (IQRs), whereas categorical variables were presented using frequencies and percentages. The chi-square test was utilized to compare differences between the two groups for categorical variables, while the t test was employed for continuous variables that followed a normal distribution. For continuous variables that did not follow a normal distribution, the rank sum test was applied. A two-tailed P value of less than 0.05 was considered statistically significant. All statistical analyses were conducted using SPSS, R, and Python software.

### Development and evaluation of predictive models for machine learning algorithms

(1) Data preprocessing. Patients with gastric cancer who received treatment at Wuxi People’s Hospital and Wuxi Second People’s Hospital between January 2010 and January 2018 were selected as the internal validation group, while patients with gastric cancer who received treatment at the Provincial Hospital affiliated with the First Medical University of Shandong Province during the same period were selected as the external validation group. The internal validation group was divided randomly into a training set (70%) and a testing set (30%). (2) The internal validation set data underwent univariate analysis, and only the variables that demonstrated significant associations were selected for the subsequent stages of the prediction model construction. (3) Build and evaluate prediction models. The selected feature variables were integrated into the prediction models of three machine learning algorithms, namely, extreme gradient boosting (XGBoost), random forest (RF), and k-nearest neighbor algorithm (KNN). Utilizing the algorithm’s underlying principle, we employ an iterative methodology to dynamically modify the model’s parameters and observe its outcomes, aiming to ascertain the model parameters that yield optimal results. To compare and select different model algorithms, k-fold cross-validation was used since it is easy to implement and has a lower bias evaluation capability compared to other methods. Hyperparameters were adjusted by grid search, and k-fold cross-validation was performed on the internal validation set using a resampling method with k=5. The dataset was divided into five groups, with one group used as a test dataset and the rest used as a training dataset. This process was repeated until each group had been used as a test dataset (Zhao et al., 2023). Model evaluation metrics such as the area under the curve (AUC), accuracy, sensitivity, and specificity were calculated and averaged over the k-round fitness to derive the most accurate estimate of the model prediction performance. The models were evaluated for discrimination, calibration, and clinical utility, and the best model was selected for prediction analysis. Receiver operating characteristic (ROC) curves were used to determine the predictive efficacy of the model, calibration curves were used to assess agreement between the predicted and actual outcomes, and decision curve analysis (DCA) was used to evaluate the clinical utility of the model. The DCA curve starts at the intersection of the red curve with the All curve and ends at the intersection of the red curve with None, within which the corresponding patient can benefit. (4) External validation of the best model will be conducted using an external test set. ROC curves will be plotted to evaluate the predictive efficiency and generalizability of the model. (5) Model interpretation. The Shapley value, obtained through Shapley additive explanation (SHAP) analysis, allows us to determine the contribution of each feature in the sample to the prediction. Based on the Shapley values, two types of plots are constructed: the SHAP summary plot, which ranks the importance of risk factors, and the single-sample SHAP force plot, which analyzes and explains the prediction results of a single sample (Chi et al., 2023).

## Results

### Basic clinical information of the patient

**Figure 1:**
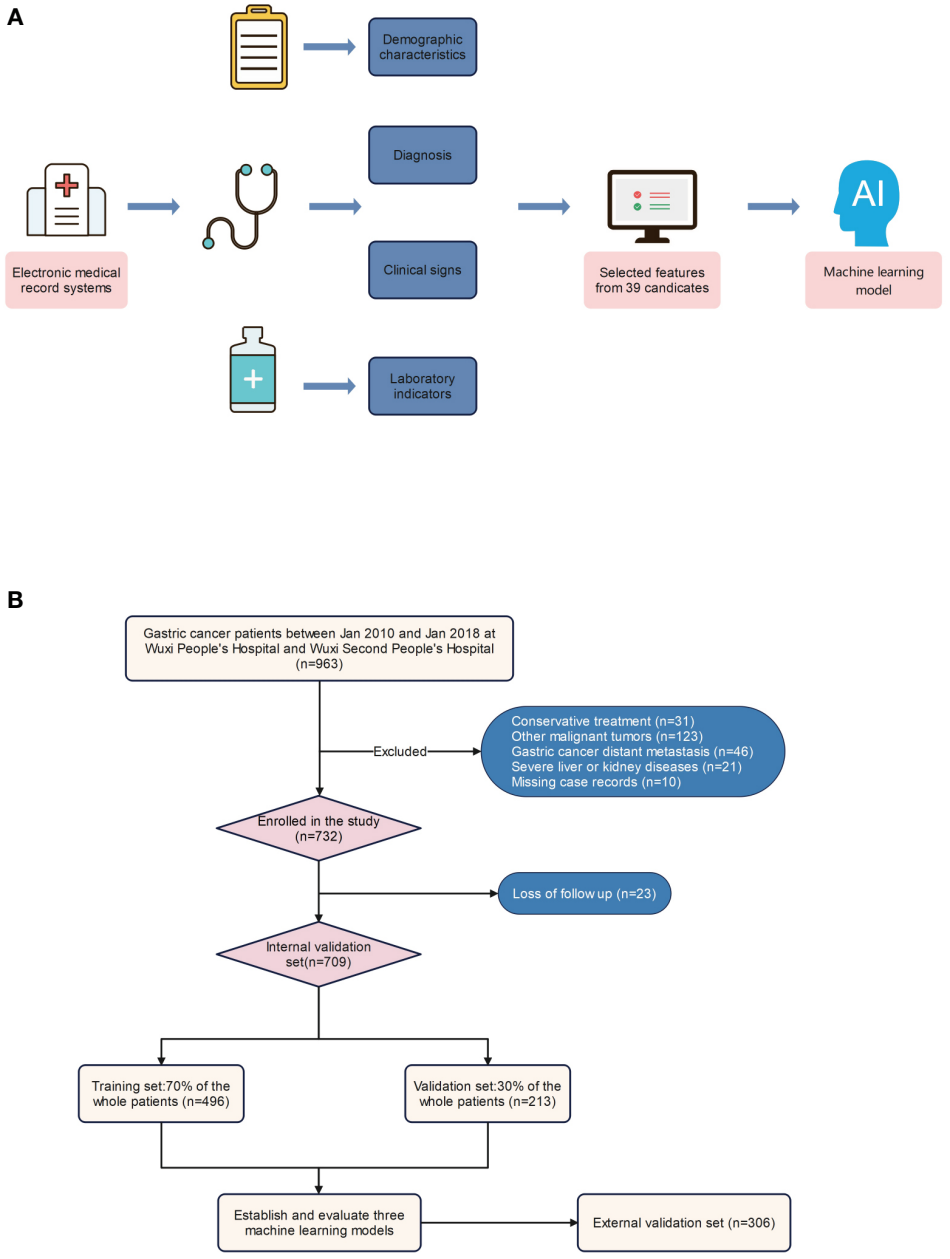
Model-making process and flowchart of the study. **(A)** Study design flow chart. **(B)** Flow diagram of patients included in the study.

A total of 1015 patients were included in the study, of whom 92 (9.06%) died within one year, 299 (29.46%) died within three years, and 404 (39.8%) died within five years (**Figures 1A, B**). The internal validation set consisted of 709 patients with gastric cancer, of whom 66 (9.31%) died within one year, 206 (29.06%) died within three years, and 281 (39.63%) died within five years. The external validation set included 306 patients with gastric cancer, of whom 26 (8.5%) died within one year, 93 (30.39%) died within three years, and 123 (40.2%) died within five years. The original data presented in the study are included in **Table S1**.

### Screening for risk factors for death at one, three, and five years after combination therapy in patients with gastric cancer

The univariate analysis results indicated that several factors significantly influenced the one-year death rate among gastric cancer patients, including age, emergency surgery, tumor T-stage, lymph node metastasis, peripheral nerve metastasis, tumor number, size, CEA level after combined treatment, CA125 level, CA72-4 level, and *H. pylori* infection (p<0.05). Similarly, the death rate at three years was influenced by several factors, such as age, surgical method, operative time, intraoperative bleeding, operation mode, tumor T-stage, lymph node metastasis, peripheral nerve metastasis, tumor number, size, CEA level, CA125 level, CA72-4 level after combined treatment, intraoperative blood transfusion, and *H. pylori* infection. Moreover, gender, age, surgical method, time of surgery, intraoperative bleeding, tumor T-stage, lymph node metastasis, peripheral nerve metastasis, tumor number, size, CEA level after combined treatment, CA125 level, CA72-4 level, NRS2002 score, intraoperative blood transfusion, and *H. pylori* infection were found to be significant influencing factors for five-year mortality in gastric cancer patients (**Table 1**).

**Table 1:** Univariate analysis of the prognosis of combined treatment.

Variables		One-year mortality		Three-year mortality		Five-year mortality	
		OR (95%CI)	P-value	OR (95%CI)	P-value	OR (95%CI)	P-value
Sex	Female	Reference		Reference		Reference	
	Male	1.029 [0.617,1.713]	0.914	0.734 [0.530,1.017]	0.063	0.567 [0.418,0.768]	<0.001
Age	<65	Reference		Reference		Reference	
	≥65	3.359 [1.995,5.656]	<0.001	4.098 [2.910,5.773]	<0.001	9.505 [6.643,13.599]	<0.001
BMI	<25 kg/m^2^	Reference		Reference		Reference	
	≥25 kg/m^2^	1.064 [0.608,1.864]	0.827	1.147 [0.801,1.641]	0.454	1.203 [0.861,1.680]	0.278
ASA	<3	Reference		Reference		Reference	
	≥3	1.155 [0.670,1.993]	0.604	0.94 [0.656,1.346]	0.736	0.927 [0.665,1.293]	0.655
Drinking history	No	Reference		Reference		Reference	
	Yes	1.052 [0.595,1.859]	0.862	0.711 [0.484,1.042]	0.081	0.855 [0.606,1.207]	0.373
Smoking history	No	Reference		Reference		Reference	
	Yes	1.634 [0.969,2.755]	0.066	1.391 [0.982,1.970]	0.063	1.368 [0.986,1.898]	0.06
ALB	≥30g/L	Reference		Reference		Reference	
	<30g/L	0.821 [0.427,1.578]	0.554	0.948 [0.635,1.415]	0.793	0.897 [0.618,1.301]	0.565
NRS2002 score	<3	Reference		Reference		Reference	
	≥3	1.33 [0.742,2.385]	0.338	0.691 [0.454,1.050]	0.083	0.634 [0.432,0.930]	0.02
Surgical history	No	Reference		Reference		Reference	
	Yes	1.452 [0.817,2.579]	0.204	1.148 [0.777,1.697]	0.489	1.133 [0.785,1.633]	0.505
Anemia	No	Reference		Reference		Reference	
	Yes	0.724 [0.377,1.389]	0.331	0.744 [0.499,1.111]	0.149	0.747 [0.518,1.076]	0.117
Hyperlipidemia	No	Reference		Reference		Reference	
	Yes	1.439 [0.791,2.615]	0.233	0.971 [0.640,1.474]	0.892	0.936 [0.635,1.378]	0.736
Hypertension	No	Reference		Reference		Reference	
	Yes	0.992 [0.586,1.680]	0.976	0.908 [0.647,1.274]	0.576	0.866 [0.632,1.185]	0.368
Diabetes	No	Reference		Reference		Reference	
	Yes	1.441 [0.782,2.656]	0.242	1.315 [0.870,1.988]	0.194	1.266 [0.856,1.873]	0.237
COPD	No	Reference		Reference		Reference	
	Yes	1.795 [0.841,3.830]	0.131	1.538 [0.891,2.653]	0.122	1.424 [0.841,2.412]	0.188
CHD	No	Reference		Reference		Reference	
	Yes	1.475 [0.670,3.246]	0.335	0.974 [0.550,1.727]	0.929	0.865 [0.506,1.479]	0.595
Adjuvant Radiotherapy	No	Reference		Reference		Reference	
	Yes	1.326 [0.731,2.406]	0.353	1.185 [0.796,1.765]	0.404	1.206 [0.830,1.752]	0.325
Adjuvant Chemotherapy	No	Reference		Reference		Reference	
	Yes	0.84 [0.453,1.558]	0.581	1.143 [0.786,1.662]	0.485	1.088 [0.766,1.546]	0.637
Surgical procedure	Laparoscopic surgery	Reference		Reference		Reference	
	Open surgery	1.18 [0.700,1.987]	0.535	0.666 [0.480,0.923]	0.015	0.671 [0.495,0.910]	0.01
Emergency surgery	No	Reference		Reference		Reference	
	Yes	1.679 [1.010,2.792]	0.046	1.05 [0.753,1.464]	0.775	1.072 [0.787,1.460]	0.659
Surgery type	Proximal gastrectomy	Reference		Reference		Reference	
	Distal gastrectomy	1.038 [0.538,2.001]	0.912	0.973 [0.656,1.444]	0.892	0.915 [0.633,1.322]	0.636
	Total gastrectomy	1.575 [0.841,2.950]	0.156	1.028 [0.686,1.540]	0.894	1.166 [0.802,1.696]	0.422
Anastomosis method	Anastomosis instruments	Reference		Reference		Reference	
	Manual anastomosis	1.717 [0.997,2.959]	0.051	1.272 [0.875,1.847]	0.207	1.247 [0.878,1.772]	0.218
Anastomosis type	Billroth I	Reference		Reference		Reference	
	Billroth II	1.271 [0.678,2.381]	0.454	1.305 [0.866,1.968]	0.203	1.165 [0.795,1.707]	0.435
	Roux-en-Y	0.955 [0.503,1.815]	0.889	1.147 [0.766,1.717]	0.505	1.264 [0.873,1.830]	0.214
Surgery time	<270 min	Reference		Reference		Reference	
	≥270 min	1.324 [0.776,2.259]	0.303	1.544 [1.092,2.183]	0.014	1.882 [1.356,2.613]	<0.001
Intraoperative bleeding	<100 ml	Reference		Reference		Reference	
	≥100 ml	1.469 [0.849,2.543]	0.169	1.864 [1.301,2.671]	0.001	2.814 [1.983,3.992]	<0.001
Blood transfusion	No	Reference		Reference		Reference	
	Yes	0.522 [0.233,1.172]	0.115	1.586 [1.059,2.377]	0.025	2.037 [1.381,3.004]	<0.001
SPO_2_	≥90%	Reference		Reference		Reference	
	<90%	1.275 [0.625,2.601]	0.504	1.283 [0.799,2.058]	0.302	1.218 [0.778,1.908]	0.388
T-stage	T1~T2	Reference		Reference		Reference	
	T3~T4	2.897 [1.733,4.843]	<0.001	2.007 [1.417,2.843]	<0.001	2.212 [1.584,3.089]	<0.001
N-stage	N0	Reference		Reference		Reference	
	N1~N3	2.289 [1.364,3.841]	0.002	2.191 [1.545,3.109]	<0.001	2.595 [1.850,3.640]	<0.001
PNI	No	Reference		Reference		Reference	
	Yes	3.908 [2.039,7.491]	<0.001	2.495 [1.461,4.259]	0.001	1.976 [1.160,3.367]	0.012
Tumor number	<2	Reference		Reference		Reference	
	≥2	3.184 [1.865,5.437]	<0.001	2.168 [1.470,3.198]	<0.001	1.853 [1.270,2.703]	0.001
Tumor size	<5 cm	Reference		Reference		Reference	
	≥5 cm	1.934 [1.103,3.392]	0.021	2.822 [1.921,4.146]	<0.001	2.539 [1.738,3.710]	<0.001
CA125 level	<35 U/ml	Reference		Reference		Reference	
	≥35 U/ml	2.691 [1.612,4.492]	<0.001	1.766 [1.255,2.487]	0.001	1.658 [1.199,2.293]	0.002
CA72-4 level	<7 U/ml	Reference		Reference		Reference	
	≥7 U/ml	2.112 [1.267,3.518]	0.004	3.036 [2.172,4.244]	<0.001	3.215 [2.341,4.415]	<0.001
CEA level	<5 ng/ml	Reference		Reference		Reference	
	≥5 ng/ml	2.414 [1.441,4.044]	0.001	1.948 [1.373,2.762]	<0.001	2.307 [1.649,3.227]	<0.001
CA19-9 level	<37 U/mL	Reference		Reference		Reference	
	≥37 U/mL	0.921 [0.516,1.641]	0.779	0.898 [0.621,1.298]	0.568	0.901 [0.641,1.266]	0.547
PCT level	<0.05 ng/ml	Reference		Reference		Reference	
	≥0.05 ng/ml	1.074 [0.601,1.919]	0.81	1.055 [0.725,1.534]	0.781	0.853 [0.599,1.214]	0.376
CRP level	<10 mg/l	Reference		Reference		Reference	
	≥10 mg/l	1.061 [0.587,1.918]	0.845	0.857 [0.580,1.268]	0.441	0.78 [0.543,1.121]	0.18
SAA level	<10 mg/l	Reference		Reference		Reference	
	≥10 mg/l	0.959 [0.486,1.890]	0.903	0.802 [0.514,1.251]	0.33	0.945 [0.633,1.411]	0.783
HP infection	No	Reference		Reference		Reference	
	Yes	2.542 [1.482,4.362]	0.001	2.751 [1.879,4.026]	<0.001	2.93 [2.006,4.278]	<0.001

OR, odds ratio; CI, confidence interval; BMI, body mass index; ASA, The American Society of Anesthesiologists; ALB, albumin; PCT, procalcitonin; CRP, C-reactive protein; SAA, serum amyloid A; NRS2002, nutrition risk screening 2002; SPO_2_, percutaneous arterial oxygen saturation; CHD, coronary heart disease; COPD, chronic obstructive pulmonary disease; PNI, peripheral nerve invasion; CEA, carcinoembryonic antigen, CA19-9, carbohydrate antigen 19-9, CA72-4, carbohydrate antigen 72-4, CA125, carbohydrate antigen 125, NLR, neutrophil-to-lymphocyte ratio.

### Model building and evaluation

**Figure 2:**
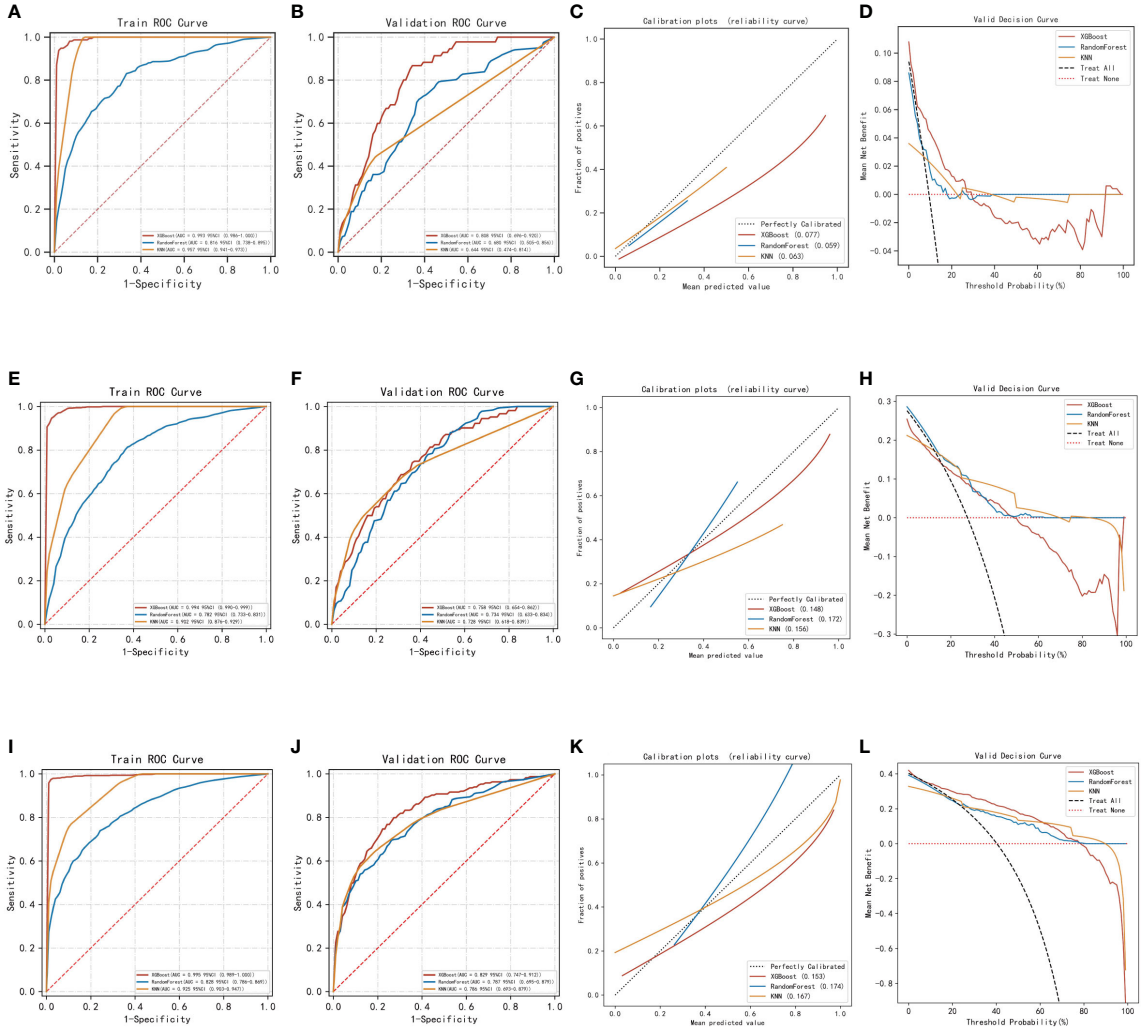
Evaluation of the three models for predicting prognosis. **(A)** ROC curves for the training set of three models predicting patient death at one year. **(B)** ROC curves for the validation set of three models predicting patient death at one year. **(C)** Calibration plots of the three models predicting patient death at one year. **(D)** DCA curves of the three models predicting patient death at one year. **(E)** ROC curves for the training set of three models predicting patient death at three years. **(F)** ROC curves for the validation set of three models predicting patient death at three years. **(G)** Calibration plots of the three models predicting patient death at three years. **(H)** DCA curves of the three models predicting patient death at three years. **(I)** ROC curves for the training set of three models predicting patient death at five years. **(J)** ROC curves for the validation set of three models predicting patient death at five years. **(K)** Calibration plots of the three models predicting patient death at five years. **(L)** DCA curves of the three models predicting patient death at five years.

Regarding the prediction analysis of one-year, three-year, and five-year death of patients with gastric cancer, the results of ROC curve analysis revealed that XGBoost had the highest performance compared to the other two algorithms. Specifically, the AUC value of XGBoost was 0.993 in the training set and 0.808 in the validation set for one-year death prediction; 0.994 in the training set and 0.758 in the validation set for three-year death prediction; and 0.995 in the training set and 0.829 in the validation set for five-year death prediction (**Table 2**). Additionally, the calibration curves of the three models were similar to the ideal curves, indicating a high level of agreement between the predicted and actual outcomes. The DCA curves also showed that all three models achieved a net clinical benefit relative to the full treatment or no treatment plan (**Figures 2A–L**). Finally, the k-fold cross-validation method was used to compare the generalization ability of the three models.

**Table 2:** Evaluation of the performance of the three models.

			AUC (95%CI)	cutoff (95%CI)	Accuracy (95%CI)	Sensitivity (95%CI)	Specificity (95%CI)	F1 Score (95%CI)
One-year mortality	KNN	training set	0.957 (0.941-0.973)	0.250 (0.250-0.250)	0.933 (0.923-0.943)	1.000 (1.000-1.000)	0.891 (0.870-0.913)	0.833 (0.767-0.900)
		validation set	0.644 (0.474-0.814)	0.250 (0.250-0.250)	0.906 (0.890-0.922)	0.416 (0.243-0.589)	0.864 (0.834-0.893)	0.346 (0.167-0.525)
	XGBoost	training set	0.993 (0.986-1.000)	0.247 (0.206-0.289)	0.969 (0.954-0.984)	0.968 (0.950-0.987)	0.970 (0.954-0.987)	0.844 (0.776-0.913)
		validation set	0.808 (0.696-0.920)	0.247 (0.206-0.289)	0.836 (0.796-0.876)	0.928 (0.864-0.991)	0.676 (0.563-0.789)	0.415 (0.297-0.533)
	RF	training set	0.816 (0.738-0.895)	0.104 (0.082-0.127)	0.775 (0.707-0.842)	0.755 (0.691-0.819)	0.775 (0.700-0.851)	0.369 (0.305-0.433)
		validation set	0.680 (0.505-0.856)	0.104 (0.082-0.127)	0.688 (0.644-0.732)	0.684 (0.386-0.982)	0.731 (0.575-0.887)	0.231 (0.157-0.305)
Three-year mortality	KNN	training set	0.902 (0.876-0.929)	0.250 (0.250-0.250)	0.831 (0.823-0.838)	1.000 (1.000-1.000)	0.663 (0.642-0.685)	0.838 (0.827-0.849)
		validation set	0.728 (0.618-0.839)	0.250 (0.250-0.250)	0.772 (0.752-0.792)	0.649 (0.515-0.782)	0.745 (0.613-0.877)	0.643 (0.541-0.745)
	XGBoost	training set	0.994 (0.990-0.999)	0.330 (0.306-0.354)	0.957 (0.944-0.970)	0.970 (0.963-0.977)	0.954 (0.932-0.975)	0.933 (0.914-0.952)
		validation set	0.758 (0.654-0.862)	0.330 (0.306-0.354)	0.712 (0.668-0.756)	0.816 (0.725-0.907)	0.614 (0.486-0.742)	0.568 (0.489-0.647)
	RF	training set	0.782 (0.733-0.831)	0.283 (0.268-0.299)	0.706 (0.678-0.734)	0.777 (0.735-0.819)	0.682 (0.632-0.731)	0.598 (0.578-0.619)
		validation set	0.734 (0.633-0.834)	0.283 (0.268-0.299)	0.676 (0.635-0.717)	0.784 (0.677-0.891)	0.627 (0.515-0.739)	0.574 (0.537-0.611)
Five-year mortality	KNN	training set	0.925 (0.903-0.947)	0.400 (0.280-0.520)	0.815 (0.790-0.840)	0.861 (0.747-0.974)	0.806 (0.696-0.915)	0.875 (0.840-0.909)
		validation set	0.786 (0.693-0.879)	0.400 (0.280-0.520)	0.750 (0.697-0.803)	0.592 (0.515-0.669)	0.877 (0.808-0.945)	0.689 (0.624-0.753)
	XGBoost	training set	0.995 (0.989-1.000)	0.414 (0.381-0.446)	0.980 (0.977-0.983)	0.977 (0.972-0.982)	0.986 (0.978-0.994)	0.978 (0.974-0.982)
		validation set	0.829 (0.747-0.912)	0.414 (0.381-0.446)	0.764 (0.751-0.777)	0.812 (0.730-0.893)	0.756 (0.690-0.822)	0.758 (0.742-0.774)
	RF	training set	0.828 (0.786-0.869)	0.416 (0.377-0.454)	0.769 (0.749-0.790)	0.721 (0.685-0.758)	0.804 (0.759-0.849)	0.716 (0.704-0.728)
		validation set	0.787 (0.695-0.879)	0.416 (0.377-0.454)	0.714 (0.665-0.763)	0.737 (0.706-0.769)	0.737 (0.680-0.793)	0.681 (0.606-0.757)

CI, confidence interval; KNN, k-nearest neighbor; XGBoost, extreme gradient boosting; RF, random forest; AUC, area under the curve.

**Figure 3:**
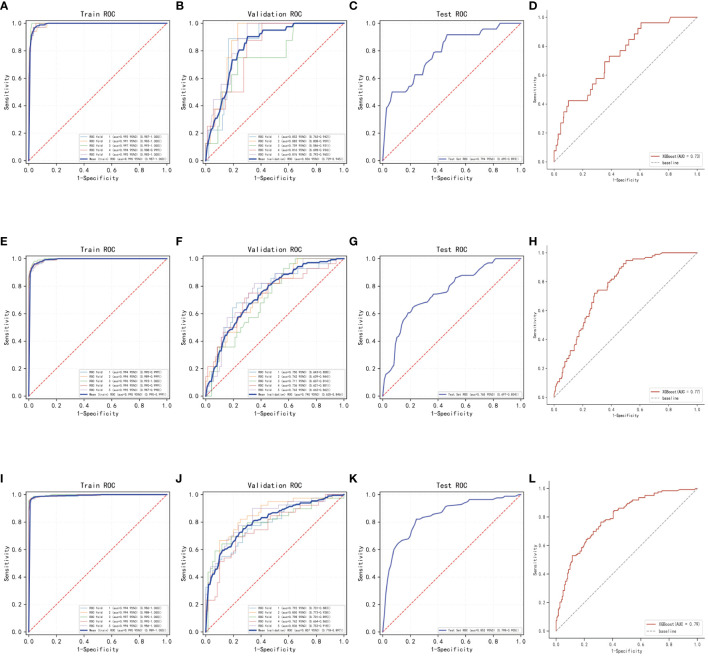
Internal validation of the XGBoost model. **(A)** ROC curves for the training set of the XGBoost model predicting patient death at one year. **(B)** ROC curves for the validation set of the XGBoost model predicting patient death at one year. **(C)** ROC curves for the test set of the XGBoost model predicting patient death at one year. **(D)** External validation of the XGBoost model predicting patient death at one year. **(E)** ROC curves for the training set of the XGBoost model predicting patient death at three years. **(F)** ROC curves for the validation set of the XGBoost model predicting patient death at three years. **(G)** ROC curves for the test set of the XGBoost model predicting patient death at three years. **(H)** External validation of the XGBoost model predicting patient death at three years. **(I)** ROC curves for the training set of the XGBoost model predicting patient death at five years. **(J)** ROC curves for the validation set of the XGBoost model predicting patient death at five years. **(K)** ROC curves for the test set of the XGBoost model predicting patient death at five years. **(L)** External validation of the XGBoost model predicting patient death at five years.

In this process, a test set comprising 213 cases (30.04%) was taken, while the remaining samples were used for training the models through 5-fold cross-validation. In the prediction of risk factors for patient mortality within one year, the XGBoost algorithm achieved an AUC of 0.8373 ± 0.0457 in the validation set and an AUC of 0.7938 in the test set, with an accuracy of 0.8873 (**Figures 3A–C**). In comparison, the RF algorithm achieved an AUC of 0.7556 ± 0.0636 in the validation set and an AUC of 0.6627 in the test set, with an accuracy of 0.6056. The KNN algorithm achieved an AUC of 0.6555 ± 0.0648 in the validation set and an AUC of 0.5787 in the test set, with an accuracy of 0.8873.

In the prediction of risk factors for patient mortality within three years, the XGBoost algorithm showed an AUC of 0.7403 ± 0.0174 in the validation set and an AUC of 0.7654 in the test set, with an accuracy of 0.7606 (**Figures 3E–G**). The RF algorithm showed an AUC of 0.6214 ± 0.0654 in the validation set, an AUC of 0.5733 in the test set, and an accuracy of 0.6808. The KNN algorithm showed an AUC of 0.7130 ± 0.0239 in the validation set and an AUC of 0.7141 in the test set, with an accuracy of 0.7183.

The results of the prediction analysis of patients’ five-year mortality showed that XGBoost had an AUC value of 0.8076 ± 0.0317 in the validation set and an AUC value of 0.8516 in the test set, with an accuracy of 0.7653 (**Figures 3I–K**). RF had an AUC value of 0.8045 ± 0.0466 in the validation set and an AUC value of 0.8089 in the test set, with an accuracy of 0.7371. KNN had an AUC value of 0.7800 ± 0.0311 in the validation set, an AUC value of 0.8297 in the test set, and an accuracy of 0.7277.

The XGBoost algorithm was selected to develop the model in this research after conducting a thorough comparison.

### Model external validation

The AUC value in the external validation set for the prediction analysis of one-year patient mortality was 0.73, for the prediction analysis of three-year patient mortality was 0.77, and for the prediction analysis of five-year patient mortality was 0.79. These values indicate that the prediction model has high accuracy in diagnosing the disease (**Figures 3D, H, L**).

### Model explanation

**Figure 4:**
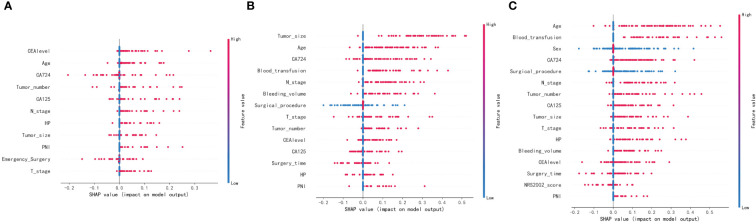
SHAP summary plot. Risk factors are arranged along the y-axis based on their importance, which is given by the mean of their absolute Shapley values. The higher the risk factor is positioned in the plot, the more important it is for the model. **(A)** SHAP summary plot of models predicting patient death at one year. **(B)** SHAP summary plot of models predicting patient death at three years. **(C)** SHAP summary plot of models predicting patient death at five years.

The SHAP summary plot results revealed that certain risk factors contribute to one-year, three-year, and five-year mortality in patients with gastric cancer. For one-year mortality, the highest-ranking risk factors were the CEA level after combined treatment, advanced age, CA72-4 level, multiple tumors, CA125 level, tumor lymph node metastasis, *H. pylori* infection, tumor size, tumor peripheral nerve metastasis, emergency surgery, and T3 and T4 tumors. The top-ranking risk factors for three-year mortality were tumor size, advanced age, CA72-4 level, intraoperative blood transfusion, tumor lymph node metastasis, intraoperative bleeding, surgical approach, tumors of T3 and T4, multiple tumors, CEA level, CA125 level, time of surgery, *H. pylori* infection, and tumor peripheral nerve invasion. For five-year mortality, the top-ranking risk factors were advanced age, intraoperative blood transfusion, sex, CA72-4 level, surgical approach, tumor lymph node metastasis, multiple tumors, CA125 level, tumor size, tumors in T3 and T4, *H. pylori* infection, intraoperative bleeding volume, CEA level, time to surgery, NRS2002 score, and peritumor nerve invasion (**Figures 4A–C**). The shared risk factors that were found to influence patient mortality at one, three, and five years after radical gastric cancer surgery included advanced age, tumors classified as T3 and T4, tumor lymph node metastasis, tumor peripheral nerve invasion, presence of multiple tumors, tumor size, elevated CEA levels, CA125 levels, CA72-4 levels, and *H. pylori* infection.

## Discussion

This study aimed to assess risk prediction models constructed using three machine learning algorithms. Of the three algorithms, XGBoost exhibited the highest accuracy (Tseng et al., 2020; Liu et al., 2023). In comparison to the RF algorithm, XGBoost employs an adaptive gradient boosting algorithm that can automatically select the optimal splitting point and tree depth, thus improving prediction performance. Furthermore, XGBoost takes into account regularization and effectively avoids model overfitting (Zhou et al., 2022). Although the KNN algorithm has higher accuracy and can avoid overfitting problems, it has high computational complexity when searching for K nearest neighbors in the training set for each test sample and calculating their distances for classification or regression prediction. Additionally, the algorithm is less stable and slower when solving problems with multiple features and large samples (DeGregory et al., 2018; Zhao et al., 2022). The XGBoost algorithm is more suitable for multidimensional studies and reduces computational effort and training time. Importantly, XGBoost provides a feature importance assessment function that can help users better understand the contribution of features in the dataset to the prediction results, improving the algorithm’s interpretability. Consequently, after a comprehensive comparison of the three machine learning algorithms, this study selected the XGBoost algorithm to construct a model to predict the long-term postoperative prognosis of gastric cancer patients.

In the realm of clinical studies, multiple risk factors may exhibit a nonlinear relationship with poor patient prognosis, particularly in the context of cancer research. This may lead to conventional models displaying suboptimal goodness of fit or limited predictive accuracy. In contrast, machine learning is capable of training algorithms to identify and discern intricate patterns, accommodating more sophisticated nonlinear relationships. As such, it may offer superiority over traditional models in medical research. Jacek et al (Baj et al., 2020). confirmed the effectiveness of machine learning algorithms for clinical diagnosis and prognosis, and this technique in artificial intelligence may also enable accurate prediction of adverse outcomes in disease progression. Notably, machine learning algorithms assumed a crucial role in developing the predictive model utilized in this study. The present model facilitates the identification of high-risk patients with precision by clinical decision-makers, enabling timely intervention to improve patient prognosis. Furthermore, it has potential utility for medical institutions to allocate resources judiciously, monitor the vital signs of high-risk patients, and enhance survival rates among gastric cancer patients.

In this study, SHAP analysis was utilized to rank the risk factors that affect the long-term prognosis of patients with gastric cancer who received combined treatment. It was discovered that *H. pylori* infection was a crucial factor among all high-risk factors. It is believed that *H. pylori* infection hinders the effectiveness of immunotherapy and promotes the growth and survival of cancer cells by altering the normal environment between the tumor and the host (Zuo et al., 2022). The primary mechanisms of action include the following: first, the infection diminishes the number of beneficial bacteria, such as lactobacilli and bifidobacteria, which has an effect on the inflammatory environment, thereby promoting the development of gastric cancer (Sipos et al., 2006). Second, *H. pylori* suppresses the activity of T cells and natural killer cells, encourages the recruitment of immunosuppressive cells, and affects the immune response in the stomach, consequently impeding the immune surveillance and clearance function of the body (Figueiredo et al., 2014). Furthermore, *H. pylori* infection is also known to stimulate the production of proinflammatory cytokines such as IL-1β, TNF-α, and IL-6, thus creating a microenvironment that promotes cancer cell growth and survival (Li et al., 2017). At the genetic level, *H. pylori* infection triggers the production of reactive oxygen species (ROS) and reactive nitrogen species (RNS), thereby increasing the risk of cancer development. Li further established a strong link between *H. pylori* and the activation of oncogenes such as c-Met and β-catenin, as well as the inactivation of tumor suppressor genes such as p53 and E-cadherin (Siregar et al., 2017), which corroborates the findings of the current study. Additionally, due to the intricate anatomy of the stomach, surgeons may find it challenging to accurately assess the extent of tumor invasion during surgery and perform intraoperative rapid pathological examination to ensure negative margins. This may result in the retention of some blood vessels of the tumor after surgery, which can become the seeds of gastric cancer recurrence, especially in patients with *H. pylori* infection. This is because *H. pylori* can accelerate the expression of vascular endothelial growth factor (VEGF), which in turn promotes the formation of new blood vessels in the tumor microenvironment, thereby promoting tumor proliferation and migration (Bagheri et al., 2018). *H. pylori* infection can also upregulate the expression of metalloproteinase-9 (MMP-9), an enzyme that degrades the extracellular matrix, which increases the risk of poor prognosis in tumor patients (Deng et al., 2022).

Similar to previous research, this study also observed that deeper tumor infiltration is correlated with an increased risk of lymphatic and peripheral nerve metastasis and a higher likelihood of postoperative mortality (Xiang et al., 2021). Highly malignant and biologically active tumor cells detach from the primary site by degrading the extracellular matrix and basement membrane *via* protein hydrolases. These detached tumor cells invade the surrounding normal tissues and enter the nearby lymph nodes. The large perigastric omentum contains numerous blood vessels, and after gastric cancer invades the surrounding lymph nodes, vascular invasion can occur, leading to the flow of tumor cells back to the liver through the portal vein system, resulting in postoperative recurrence or metastasis (Oster et al., 2022). Furthermore, these tumors can metastasize to retroperitoneal organs *via* lymph nodes, and clinical manifestations are often obscure, with imaging examinations being difficult to diagnose. David’s study (Shibata et al., 2002) also demonstrated a strong correlation between lymph node metastasis and poor outcomes in patients with tumors, while Radespiel (Radespiel-Tröger et al., 2004; Turgeon et al., 2021) discovered that the higher the number of lymph node metastases, the greater the chance of tumor recurrence and the higher the postoperative mortality rate. This emphasizes the importance of thoroughly removing relevant lymph nodes during radical surgery for gastric cancer while avoiding compression of the tumor to prevent dissemination into the abdominal cavity.

Furthermore, the size of tumors has been shown to have a significant impact on patient prognosis. We hypothesize that larger tumors have a higher proliferation rate and generate more tumor vessels. Tomisaki conducted a study on 175 patients with gastrointestinal tumors and found a strong correlation between metastatic recurrence and microvessel density (MVD). The higher the MVD, the greater the likelihood of tumor cells entering the circulatory system (Tomisaki et al., 1996). Similarly, Park reported that larger tumors have a higher risk of shedding tumor cells into the abdominal and pelvic cavities and vascular tissues, thus increasing the potential risk of tumor recurrence after surgery (Park et al., 1999). Multiple gastric cancers also pose a challenge for treatment and are associated with a higher risk of tumor recurrence. Surgical removal of the primary tumor may reduce the concentration of tumor growth inhibitory factors and accelerate residual tumor recurrence. Li et al. investigated this hypothesis using two groups of mouse models, with the experimental group undergoing conventional tumor resection and the control group undergoing sham surgery. The results showed significant differences in tumor growth and recurrence between the experimental and control groups (Li et al., 2001).

The findings of the current investigation suggest that patients who display elevated levels of CEA after undergoing radical gastrectomy for gastric cancer, when followed up with immunotherapy, are at an increased risk of mortality. Tsuyoshi previously identified CEA as an acidic glycoprotein expressed by normal human mucosal cells that lacks specificity in detecting gastrointestinal tumors (Konishi et al., 2018). However, with the advancement of medical diagnostic techniques in recent years, clinicians have recognized the clinical significance of CEA. Polat conducted a prospective study to explore the association between serum levels of tumor markers and clinical variables in patients with gastrointestinal tumors. In a subsequent investigation, Tsuyoshi et al. demonstrated that most patients’ serum CEA levels returned to normal three months after combination therapy, while another group of patients with persistent CEA elevation after treatment had a rapid recurrence of tumors compared to their counterparts with normal posttreatment CEA levels. The results of the present study indicate that an increase in CEA levels after gastric cancer surgery could be indicative of tumor recurrence (Attallah et al., 2018; Konishi et al., 2018). Recently, some clinicians have employed a combination of CEA, CA19-9, cytokeratin-1 (CK-1), CA72-4 and mucin-1 (MUC-1) to predict unfavorable outcomes in gastrointestinal tumors, which has improved the sensitivity and specificity of tumor surveillance while also evaluating tumor stage and metastasis (Pua et al., 2020).

In recent years, medical practitioners have endeavored to utilize certain tests to prognosticate the outcomes of immunotherapy in conjunction with surgical interventions. However, it has been observed that such approaches exhibit a higher rate of misdiagnosis and fail to significantly influence patient prognoses. Consequently, we have opted to employ more precise machine learning algorithms for the purpose of identifying high-risk factors and enhancing patient prognoses. The present study provides a comprehensive evaluation of the model in terms of discrimination, calibration, and clinical utility, yet certain limitations exist. While the study accounted for multiple aspects of risk factors, imaging-related aspects were not considered. Furthermore, while the machine learning algorithms were more accurate, their models were more intricate and less transparent. The entire computational and decision-making process of the model is opaque, lacking the intuitive and clear features of the logistic regression model. Conversely, this retrospective study suffers from selection bias, distribution bias, and retrospective bias. Thus, further international, multicenter, large-scale studies are necessary to validate the reliability of our findings.

## Conclusion

This study presented the development of a prediction model utilizing the XGBoost machine learning algorithm to assess the risk of mortality in tumor patients who underwent radical gastric cancer surgery along with immunotherapy. The model demonstrated promising accuracy and clinical value, enabling surgeons to diagnose patients promptly. The model identified that a negative outcome in gastric cancer patients correlated with various factors, including older age, tumor invasion, tumor lymph node metastasis, peripheral nerve invasion, presence of multiple tumors, larger tumor size, increased levels of CEA, CA125, and CA72-4, and *H. pylori* infection.

## Data availability statement

The original contributions presented in the study are included in the article/**Supplementary Material**. Further inquiries can be directed to the corresponding authors.

## Ethics statement

The studies involving human participants were reviewed and approved by the Ethics Committee of the Affiliated Wuxi People’s Hospital of Nanjing Medical University, Wuxi Second People’s Hospital, and Shandong Provincial Hospital affiliated with Shandong First Medical University. The patients/participants provided their written informed consent to participate in this study. Written informed consent was obtained from the individual(s) for the publication of any potentially identifiable images or data included in this article.

## Author contributions

YL and WS conceived the study. YL, WD, YH and LW drafted the manuscript. YG, CS, SN, JX and ZT analyzed and visualized the data. CC, JL and WS helped with the final revision of this manuscript. All authors contributed to the article and approved the submitted version.

## Supplementary material

Supplementary Table 1 Raw data.
